# Regeneration enhancers: A clue to reactivation of developmental genes

**DOI:** 10.1111/dgd.12654

**Published:** 2020-02-25

**Authors:** Nanoka Suzuki, Haruki Ochi

**Affiliations:** ^1^ Amphibian Research Center Hiroshima University Higashi‐Hiroshima Japan; ^2^ Institute for Promotion of Medical Science Research Faculty of Medicine Yamagata University Yamagata Japan

**Keywords:** animals, developmental genes, regeneration, transcription factors, *Xenopus laevis*

## Abstract

During tissue and organ regeneration, cells initially detect damage and then alter nuclear transcription in favor of tissue/organ reconstruction. Until recently, studies of tissue regeneration have focused on the identification of relevant genes. These studies show that many developmental genes are reused during regeneration. Concurrently, comparative genomics studies have shown that the total number of genes does not vastly differ among vertebrate taxa. Moreover, functional analyses of developmental genes using various knockout/knockdown techniques demonstrated that the functions of these genes are conserved among vertebrates. Despite these data, the ability to regenerate damaged body parts varies widely between animals. Thus, it is important to determine how regenerative transcriptional programs are triggered and why animals with low regenerative potential fail to express developmental genes after injury. Recently, we discovered relevant enhancers and named them regeneration signal‐response enhancers (RSREs) after identifying their activation mechanisms in a *Xenopus laevis* transgenic system. In this review, we summarize recent studies of injury/regeneration‐associated enhancers and then discuss their mechanisms of activation.

## Graphical abstract

Summary of recent studies of injury/regeneration‐associated enhancers and their activation mechanisms.

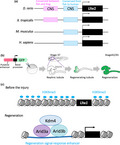

## 1 INTRODUCTION

The ability to regenerate lost or damaged body parts is widespread among animals, although the extent of this ability varies (Tanaka & Reddien, 2011). Amphibians and fish can regenerate numerous tissues, whereas mammals have limited regenerative capacity (Poss, 2010; Tanaka & Reddien, 2011). In kidney tissues, the nephron functional unit does not differ much among vertebrates (Lienkamp, 2016). The nephron comprises a filtering component known as the glomerulus and a nephric tubule, which is divided into the following four basic domains: proximal tubule, loop of Henle, distal tubule, and connecting tubule (Lienkamp, 2016). Mammalian nephrons have cells that contribute to repair after injury, yet their regenerative capacity is limited to nephric epithelial cells in damaged regions (Maeshima, Nakasatomi, & Nojima, 2014). In contrast, the African clawed frog (*Xenopus laevis*) and zebrafish (*Danio rerio*) regenerate fully coiled and functional nephric tubule architectures after severe damage (Caine & Mclaughlin, 2013; Diep et al., 2011).

During tissue and organ regeneration, cells respond to damage by stimulating proliferation, differentiation, and developmental patterning (Brockes & Kumar, 2008; Poss, 2010; Tanaka & Reddien, 2011), resembling developmental processes except in processes such as wound healing, dedifferentiation, and transdifferentiation (Brockes & Kumar, 2008; Poss, 2010; Tanaka & Reddien, 2011; Iismaa et al., 2018). Molecular studies of regeneration have identified many developmental genes that are activated during regeneration. These studies also show that genes involved in regeneration are frequently conserved among vertebrates, although a few genes such as *Prod1* and *Ag1* (*Anterior gradient*) have been reported as species‐specific genes (Da Silva, Gates, & Brockes, 2002; Ivanova, Tereshina, Ermakova, Belousov, & Zaraisky, 2013). Prod1 was first identified as a newt (*Notophtalmus viridescens*) ‐specific ortholog of CD59 that is expressed in blastemas, which are growth zones of mesenchymal stem/progenitor cells. Moreover, transcription activator‐like effector nuclease (TALEN)‐mediated gene knockout of F0 salamanders (*Pleurodeles waltl*) showed that Prod1 is involved in both limb development and regeneration (Da Silva et al., 2002; Kumar, Gates, Czarkwiani, & Brockes, 2015). Whole‐genome sequencing analyses of the Mexican axolotl (*Ambystoma mexicanum*) with other large genome sequences identified *Prod1* as a member of the lymphocyte antigen 6 (Ly6)/urokinase‐type plasminogen activator receptor (uPAR) family rather than as a homolog of CD59 (Nowoshilow et al., 2018). Human and mouse genomes contain 35 and 65 Ly6/uPAR family members, respectively, and share characteristic domains, such as the LU domain (Loughner et al., 2016). Ly6/uPAR proteins have a wide range of functions during cell proliferation, migration, cell–cell interactions, immune cell maturation, macrophage activation, and cytokine production. These functions may also be conserved in newts, salamanders, and axolotls. Ag1 (nAG) is a homolog of secreted *Xenopus laevis* xAgr1 and xAgr2 proteins, and was identified as a ligand of Prod1 in a yeast two‐hybrid screen (DePamphilis, Gray, & Trost, 2007). Comparative genomics analyses also show that *Ag1*, with *Agr2* and *Agr3*, comprises the superfamily of protein disulphide isomerases, although *Ag1* is no longer present in mammals, birds, and reptiles (Ivanova et al., 2013). The continued presence of Ag1 in fish and amphibian genomes suggests that its absence in other species is related to the loss of appendage regeneration (Ivanova et al., 2013). However, previous studies show interactions between human Agr2 and Agr3 proteins and Ly6/PLAUR domain containing 3 (LYPD3/C4.4a) that resemble Ag1–Prod1 interactions, suggesting evolutionary conservation of Ag1/Agr2/Agr3 and Prod1/Ly6 mechanisms among vertebrates (Fletcher et al., 2003; Loughner et al., 2016). These findings imply that regenerative capacity cannot be related to the presence or absence of specific genes in the genome. Alternatively, gene regulatory mechanisms may better reveal the molecular basis of regeneration after injury. To our knowledge, no extensive analyses indicate whether or to what degree regenerative animals use unique *cis*‐regulatory elements to induce developmental genes during regeneration.

In recent years, *cis*‐regulatory elements that are involved in injury and/or regeneration have been identified in several model animals (Mead et al., 2013; Kang, Karra, Dickson, Nachtrab, & Goldman, 2016; Harris, Setiawan, Saul, & Hariharan, 2016; Rodriguez & Kang, 2019; Yang & Kang, 2019). In addition, epigenetic modifications of enhancers that are strongly implicated in gene expression have been reported. In this review, we summarize similarities between development‐ and regeneration‐related genes and provide an overview of recent studies of injury/regeneration‐associated enhancers and their mechanisms of activation.

## 2 EVOLUTIONARILY CONSERVED REGENERATION‐ASSOCIATED GENES

Signaling pathways involving proteins of int1/Wingless (Wnt), fibroblast growth factor (Fgf), transforming growth factor β (Tgf‐β), Hedgehog (Hh), and Notch families have been associated with tissue and organ development, and many of these also contribute to regeneration. In particular, Wnt/β‐catenin signaling is necessary for the development of various tissues and stem cells, and genes that encode components of this signaling pathway are evolutionarily conserved among animals (Freese, Pino, & Pleasure, 2010; Clevers, Loh, & Nusse, 2014). Dickkopf1 (Dkk1) is a secreted antagonist of Wnt/β‐catenin signaling. Induction of Dkk1 expression using a heat shock‐inducible transgenic system immediately before limb amputation prevented limb regeneration in *X. laevis* tadpoles (Yokoyama, Ogino, Stoick‐Cooper, Grainger, & Moon, 2007). Similarly, heat shock induction of Dkk1 expression in transgenic *X. laevis* tadpoles led to failure of tail regeneration (Lin & Slack, 2008). Conversely, glycogen synthase kinase 3β (GSK‐3β) is a negative regulator of Wnt/β‐catenin signaling, and treatments with the specific GSK‐3β inhibitor BIO promoted tail outgrowth (Lin & Slack, 2008).

Fgf and Tgf‐β are required for the development of various tissues and are also known to control tissue regeneration of axolotl and *X. laevis* tadpole limbs and chicken (*Gallus gallus*) and zebrafish retinas (Lévesque et al., 2007; Ho & Whitman, 2008; reviewed by Maddaluno et al., 2017) . Inhibition of Fibroblast growth factor receptor (Fgfr) and Kinase insert domain receptor (Kdr, alias name: vascular endothelial growth factor receptor2 (Vegfr2)) by SU5402 reduced cell proliferation and prevented the formation of blastemas during *X. laevis* tadpole tail regeneration (Lee, Grill, Sanchez, Murphy‐Ryan, & Poss, 2005; Whitehead, Makino, Lien, & Keating, 2005; Lin & Slack, 2008). Moreover, blastema formation was arrested in a temperature‐sensitive *fgf20a* zebrafish mutant at non‐permissive temperatures (Whitehead et al., 2005). In addition, heat shock‐dependent dominant‐negative Fgfr1 expression led to failure of caudal fin regeneration in zebrafish (Lee et al., 2005). Fgf and/or Vegfr signaling may also be involved in transdifferentiation, because SU5402 treatments prevented the differentiation of iris pigment epithelial cells into lens cells in newts (Del Rio‐Tsonis, Trombley, McMahon, & Tsonis, 1998). Similarly, treatment with the TGF‐β type I receptor inhibitor SB‐431542 suppressed cell proliferation and caused failure of regeneration during axolotl limb regeneration (Lévesque et al., 2007).

The receptor mediated extracellular signals mentioned above regulate gene expression through transcription factors (TFs) that are evolutionarily conserved among animals. In particular, the TF c‐Jun is a component of AP‐1 and regulates many genes that are involved in proliferation and cell cycle progression. Transgenic analysis in Nestin‐Cre mice that express Cre recombinase in neural stem cells and intermediate neural progenitor cells showed that c‐Jun regulates axonal regeneration in mice (Raivich et al., 2004). In addition, Sox family TFs are found in all animals. Among these, Sox11 is expressed in central and peripheral nervous systems, and is induced after axonal injury (Struebing et al., 2017). Injections of *Sox11* targeted small interfering RNA (siRNA) into mouse dorsal root ganglion (DRG) neurons caused transient knockdown of *Sox11* mRNA (Jankowski et al., 2009). These investigators showed that regeneration of DRG neurons following nerve cut injury was associated with increased *Sox11* transcription and *Sox11* siRNA accordingly prevented regeneration (Jankowski et al., 2009). In a similar study, spinal cord‐specific knockdown of Sox2 was achieved using electroporation of antisense morpholino oligonucleotide into *X. laevis* tadpoles. These authors concluded that Sox2 is required for recovery of axon trajectories after spinal cord injury (Muñoz et al., 2015). The basic helix‐loop‐helix (bHLH) TF Hand2 is known to regulate heart development in early embryos (Barnes & Firulli, 2009). Heat shock induction of Hand2 expression in transgenic zebrafish enhanced cardiomyocyte proliferation during regeneration, although whether Hand2 is required for heart regeneration remains unclear (Schindler et al., 2014). T‐box TF Tbx5 is known to regulate heart development, and conditional inactivation of Tbx5a in Cre recombinase‐inducible transgenic zebrafish impaired heart regeneration (Grajevskaja, Camerota, Bellipanni, Balciuniene, & Balciunas, 2018). In addition, the Yes‐associated protein (YAP) transcriptional coactivator with the DNA‐binding TF TEAD were identified as downstream effectors of the Hippo pathway that modulates cell proliferation, differentiation, and growth (Yu & Guan, 2013). In *X. laevis* tadpoles, overexpression of dominant‐negative YAP in tadpole limbs reduced cell proliferation and led to failure of regeneration (Hayashi, Tamura, & Yokoyama, 2014). The regenerative TFs summarized above are evolutionarily conserved among vertebrates, further suggesting that most vertebrates, including humans, have an intrinsic ability to regenerate lost or damaged body parts. These observations also imply that regenerative capacities are generally not directly related to the presence or absence of specific genes.

## 3 EPIGENETIC REGULATION IN VERTEBRATE REGENERATION

Epigenetic modification of DNA and histones is an essential regulator of gene expression. Active enhancers are often correlated with histone H3 lysine 27 acetylation (H3K27ac) and actively transcribe RNA polymerase II (Pol II), whereas inactive enhancers have been correlated with histone H3 lysine 27 trimethylation (H3K27me3) and H3K9 di‐ and trimethylation (H3K9me2/3; Fischle et al., 2003; Andersson et al., 2014).

The lysine demethylase 6B/Jmjd3 (Kdm6b) and the lysine‐specific demethylase 6A/Utx (Kdm6a) are demethylates for H3K27me3, which are associated with transcriptional silencing. After injecting Kdm6b.1‐specific antisense morpholino oligonucleotides into zebrafish embryos at the one‐cell stage and amputating caudal fins at 48–72 hr, Stewart, Tsun, & Belmonte, 2009 showed that H3K27 me3 demethylase is required for caudal fin regeneration (Stewart et al., 2009). A system of tactile sense organs, known as the lateral line, comprises neuromasts that are distributed along the head and body surface (Harris et al., 2003). Neuromasts contain hair cells that are similar to sensory hair cells in mammalian inner ears, and zebrafish lateral line hair cells can regenerate after neomycin damage (Harris et al., 2003). Pharmaceutical analyses showed that treatment with the selective Kdm6b and kdm6a inhibitor GSK J4 suppresses cell proliferation in regenerating neuromasts (Bao, He, Tang, Li, & Li, 2017). Enhancer of zeste 2 polycomb repressive complex 2 subunit (Ezh2) is the catalytic subunit of Polycomb repressive complex 2 (PRC2), which is a highly conserved histone methyltransferase that targets H3K27. Treatment with the Ezh2 inhibitor 3‐deazaneplanocin A (DZNep) prevented regeneration of amputated *X. laevis* tadpole limbs (Hayashi, Kawaguchi, Uchiyama, & Kawasumi‐kita, 2015). In addition, a mutant version of histone 3 (H3.3K27M), in which the lysine (K) at position 27 was substituted for methionine (M), also had decreased H3K27me3 modifications and modest increases in H3K27ac modifications (Lewis et al., 2013; Ben‐Yair et al., 2019). Specific expression of H3.3K27M in cardiomyocytes during regeneration reduced the expression of sarcomere and cytoskeletal genes in proliferative cardiomyocytes following cardiac injury in zebrafish (Ben‐Yair et al., 2019). These data clearly indicate that gene silencing occurs during heart regeneration. Thus, H3K27me3‐related demethylases and methyltransferases contribute to regeneration.

Enrichment of H3K9me3 is often observed in heterochromatic regions, and is integral to establishing and maintaining cell fates (Becker, Nicetto, & Zaret, 2016). In this context, H3K9 methylation blocks induction of pluripotent stem cells (iPSCs) during fibroblast reprogramming, and H3K9me3 impedes the establishment of the totipotent state from mammalian oocytes through somatic cell nuclear transfer (Chen et al., 2013; Matoba et al., 2014). Thus, H3K9me3‐modified heterochromatin is present at lower levels in embryonic stem cells than in differentiated cells.

Histone acetylation plays a pivotal role in regeneration and is regulated by the balance of histone acetyltransferase (HAT) and histone deacetylase (HDAC) activities (Seto & Yoshida, 2014). Transcripts of HDAC1 are present during *X. laevis* tadpole tail regeneration (Tseng, Carneiro, Lemire, & Levin, 2011). Moreover, pharmacological inhibition of HDACs using trichostatin A (TSA) increased acylation of histone H4, and inhibited tail regeneration (Tseng et al., 2011). In another study, matured retinal ganglion cells failed to regenerate axons following optic nerve damage in mice, yet adenoviral overexpression of the histone acetyltransferase p300 promoted axonal regeneration after crushing of the optic nerve (Gaub et al., 2011). TSA treatments also reportedly led to the induction of multiple regeneration‐associated genes and promoted sensory axon regeneration after spinal cord injury in mice (Finelli, Wong, & Zou, 2013). Thus, appropriate histone acetylation status is essential for regeneration.

DNA methylation at enhancers and promoters are known to be associated with transcriptional repression, and DNA methyltransferases (DNMTs) are involved in establishing DNA methylation status (Li & Zhang, 2014). Three major DNMTs have been identified to date. DNMT1 maintains DNA methylation patterns during cell division, whereas DNMT3a and DNMT3b are essential for de novo methylation (Li & Zhang, 2014). The protein Shh is known to regulate limb development, and its expression is driven by a limb specific enhancer known as mammals‐fishes‐conserved‐sequence 1 (MFCS1; Visel, Rubin, & Pennacchio, 2009). It is known that *X. laevis* tadpoles can regenerate limbs completely, whereas as young adults after metamorphosis, *X*. *laevis* froglets regenerate only simple cartilaginous spike structures without digits after limb amputation. Analyses of DNA methylation statuses showed that MFCS1 is hypomethylated in *X. laevis* tadpoles and is subsequently highly methylated in froglets, suggesting that methylation of MFCS1 inhibits regenerative capacity (Yakushiji et al., 2007). Extensive analyses using specific inhibitors of DNMTs and/or knockout/knockdown techniques are required to confirm this hypothesis (Yakushiji et al., 2007). In transgenic zebrafish specifically expressing nitroreductase in pancreatic β‐cells under the control of the insulin promoter, treatments with metronidazole (MZT) caused β‐cell ablation (Curado et al., 2007). Although β‐cell‐ablated wild‐type zebrafish regenerated β‐cells within 48 hr after washout of MZT, *Dnmt1* mutant zebrafish exhibited significantly greater numbers of regenerated β‐cells than wild‐type zebrafish (Anderson et al., 2009). Hence, surviving pancreatic cells in *Dnmt1* mutant zebrafish may have an increased capacity to differentiate into β‐cells (Anderson et al., 2009). Perhaps appropriate DNA methylation patterns are essential for regeneration.

Multiple studies show that histone and DNA methylation levels are involved in declining regenerative capacities with maturation. Consequently, regeneration may require appropriate histone methylation, histone acetylation, and DNA methylation statuses.

## 4 ENHANCERS ARE KEY REGULATORS OF GENE EXPRESSION

Noncoding DNA regions have various known functions (Ong & Corces, 2011). Among them, enhancers, silencers, and promoters control gene expression and are referred to as *cis*‐regulatory elements. Promoters are frequently located near transcription initiation sites, and recruit general TFs to achieve basal transcription levels (reviewed by Ong & Corces, 2011). In contrast, enhancers and silencers are located proximally and distally to gene bodies. These elements control cell‐ and tissue‐specificity of gene expression, and the timing and quantity of respective transcripts (reviewed by Cho, 2012; Ong & Corces, 2011). Therefore, enhancers and silencers are critical for normal tissue and organ development, appropriate responses to environmental conditions, and maintenance of physiological conditions.

## 5 IDENTIFICATION OF ENHANCERS

Current estimates suggest that hundreds of thousands of enhancers are present in the human genome, far exceeding the number of genes (Dunham et al., 2012; Fishilevich et al., 2017). Some of these noncoding sequences are evolutionarily conserved from fish to humans and function as developmental enhancers (McEwen et al., 2009). Species‐specific enhancers have also been identified (Prescott et al., 2015; Sasaki et al., 2008). Yet, because these enhancers are involved in many biological phenomena, including regeneration, methods for identifying enhancers and analyzing their functions are key to the understanding of how developmental genes are reused after injury.

**Figure 1:**
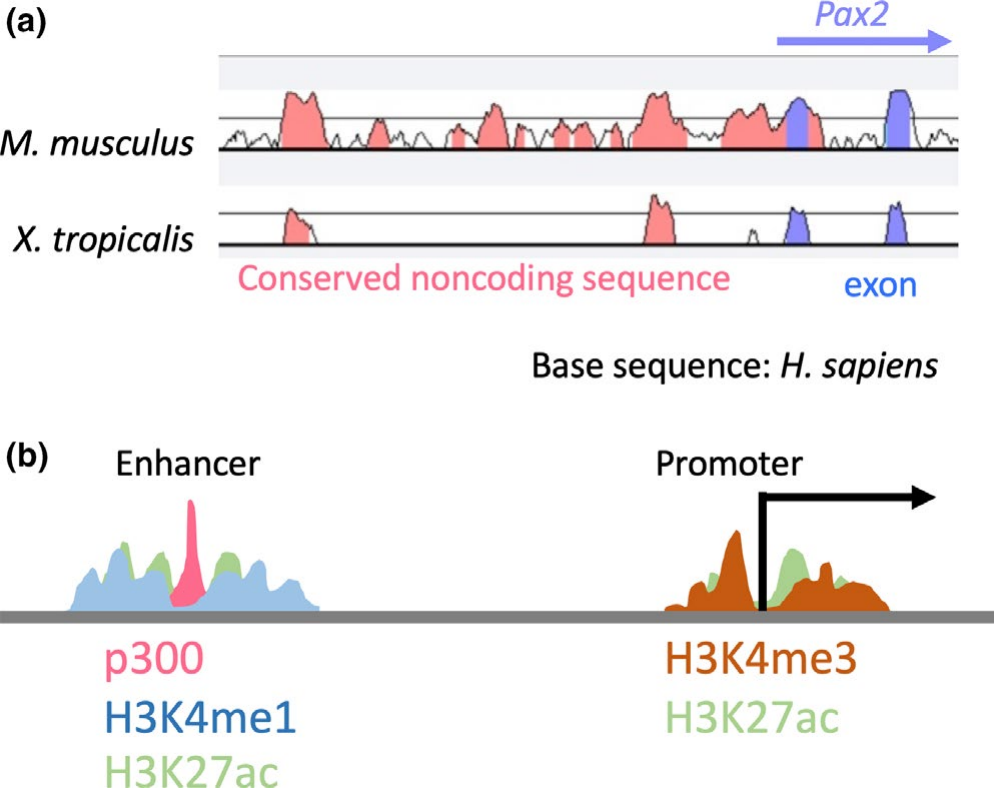
(a) Extraction of candidate enhancers using evolutionarily conserved noncoding sequences; plot using the comparative genomics alignment tool VISTA; comparison of human (*Homo sapiens)*, mouse (*Mus musculus*), and frog (*Xenopus tropicalis*) *Pax2* loci; the pink peak indicates the conserved noncoding sequences and the blue peak indicates exons. (b) Extraction of candidate enhancers based on epigenetic profiling of H3K4me1, H3K4me3, and H3K27ac, and the binding of histone acetyltransferase p300; H3K4me1, H3K27ac, and p300 are often associated with enhancers, whereas H3K4me3 and H3K27ac are often present at active promoters. The image was adapted and modified from Prescott et al. (2015)

Before the completion of the human genome project, scientists had to clone genomic fragments individually to examine their enhancer activities in cultured cells (Goto, Okada, & Kondoh, 1990; Matsuo, Kitamura, Okazaki, & Yasuda, 1991). Following the human genome project, whole‐genome sequencing of many species and the encyclopedia of DNA elements (ENCODE) project have provided novel approaches for identifying putative enhancers. Specifically, comparisons of whole‐genome sequences across species can be used to identify evolutionarily conserved sequences in noncoding genomic regions. These are known as conserved noncoding elements or conserved noncoding sequences (CNS; McEwen et al., 2009; Poliakov, Foong, Brudno, & Dubchak, 2014; Figure 1a). CNSs are often associated with enhancers for tissue and organ development (McEwen et al., 2009; Ochi et al., 2012). Researchers of the ENCODE project have mapped regions with histone modifications, chromatin structures, and TF associations (Feingold et al., 2004). Moreover, profiling of histone markers among different cell lines using chromatin immunoprecipitation (ChIP) sequencing revealed that H3K4me1 and H3K27ac are predominant histone modifications at nucleosomes flanking enhancer elements, whereas H3K4me3 H3K27ac are often present at active promoters (Liu & Hauser, 2007; Calo & Wysocka, 2013; Prescott et al., 2015; Figure 1b). ChIP‐sequencing of the transcription coactivator p300/CBP also showed that p300/CBP enrichment is often associated with enhancers (Visel, Blow, et al., 2009; Figure 1b). Open chromatin regions were also identified in deoxyribonuclease I (DNase I) sequencing (DNAse‐seq) analyses, and putative enhancers can be identified using transposase‐accessible chromatin sequencing (ATAC‐seq) analyses (Buenrostro, Giresi, Zaba, Chang, & Greenleaf, 2013; Sabo et al., 2004). Although these methods can be used to identify putative enhancers, further studies are required to determine whether they genuinely act as enhancers, and under which spatiotemporal conditions. Although transgenic techniques can reveal *in vivo* activities of enhancers, generating transgenic animals is resource‐intensive and laborious (Ogino, Ochi, Uchiyama, Louie, & Grainger, 2012). Therefore, only after narrowing down putative enhancers, based on genome‐wide information, is it feasible to validate enhancer activities using transgenic animals.

Many functional enhancers have been identified using the combination of genome‐wide information and transgenic assays. However, most are associated with tissue and organ development and few have been shown to contribute to regeneration (reviewed by Yang & Kang, 2019). Developmental enhancers can be identified by examining reporter gene expression during specific developmental stages. Yet, regeneration enhancers are inactive in uninjured tissues, and confirmation of injury/regeneration‐responses of candidate enhancers requires the use of transgenic reporter animals and additional regeneration assays. Nonetheless, efforts to identify injury‐ and regeneration‐associated enhancers are discussed in the following sections.

## 6 INJURY/REGENERATION‐RESPONSE ENHANCERS IN *DROSOPHILA*

**Figure 2:**
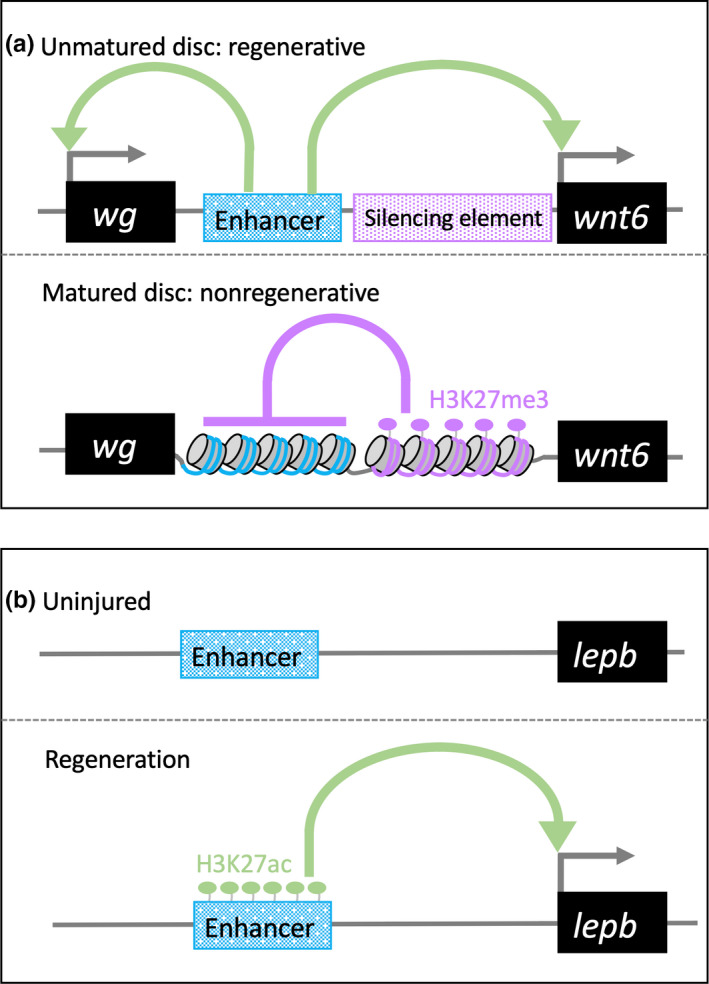
(a) Regenerative mechanisms of the damage response enhancer for *Drosophila* wing imaginal discs; *Wg* and *Wnt6* expression are upregulated in response to damage via the damage response enhancer in unmatured discs. In contrast, immediately adjacent regulatory elements promote methylation of H3K27me3 across the Wnt gene cluster. This methylation event prevents regeneration of wing imaginal discs. The image was adapted and modified from Harris et al., (2016). (b) Genomic DNA regions surrounding the *lepb* gene and profiles of H3K27ac in uninjured and regenerating hearts; transgenic analyses showed that H3K27ac‐rich elements have enhancer activity in regenerating zebrafish hearts

The fruit fly *Drosophila melanogaster* is a genetic model system that has been used to study a broad range of phenomena for over a century. Genetic screens of *Drosophila* have identified multiple developmental genes, and gene knockout/knockdown analyses in vertebrates have revealed functional conservation of developmental gene homologs in *Drosophila* and vertebrates (Jennings, 2011). Moreover, genetic studies using *Drosophila* imaginal discs have provided important insights into tissue development and regeneration, and reveal the molecular mechanisms of enhancers (Schubiger, Sustar, & Schubiger, 2010; Harris et al., 2016). *Drosophila* imaginal discs are known to regenerate following genetic ablation by inducing apoptosis in disc cells, but the capacity for regeneration is diminished during the later stages of the third larval instar (L3), when larvae approach the onset of metamorphosis (Smith‐Bolton, Worley, Kanda, & Hariharan, 2009). The Wnt1 ortholog *wingless* (wg) is upregulated in regenerating discs following ablation of wing imaginal discs (Smith‐Bolton et al., 2009). Yet in matured discs, which do not regenerate, *wg* expression is not induced (Smith‐Bolton et al., 2009). In examinations of genomic DNA fragments spanning the entire Wnt gene cluster, a 3‐kb region between *Wg* and *Wnt6* genes had enhancer activity in imaginal discs following injury. The authors accordingly referred to the region as a damage response enhancer (Schubiger et al., 2010; Harris et al., 2016; Figure 2a). Consistent with declines in regenerative capacity of wing discs and failure of *wg* expression with maturation, H3K27me3 levels adjacent to this damaged response enhancer were increased (Harris et al., 2016; Figure 2a). This epigenetic modification reportedly suppressed enhancer activity in matured discs and prevented *wg* expression in response to injury of matured discs (Harris et al., 2016; Figure 2a). Thus, epigenetic regulation of injury/regeneration‐associated enhancers governs enhancer activities before and after injury.

## 7 INJURY/REGENERATION‐ASSOCIATED ENHANCERS IN VERTEBRATES

In the past few years, *cis*‐regulatory elements involved in injury and/or regeneration have been identified in several model animals. These enhancers are known as damage response enhancers, injury response enhancers, wound induced enhancers, and regeneration enhancers (reviewed in Rodriguez & Kang, 2019). Regeneration is understood according to regeneration‐specific processes such as wound healing, dedifferentiation, and transdifferentiation, and subsequent processes that are also developmental, such as proliferation, morphological patterning, and differentiation. As discussed above, transgenic animals are required to validate enhancer activities, but current technologies do not distinguish between injury‐specific and regeneration‐specific types. Therefore, we refer to enhancers that are involved in regeneration as injury/regeneration‐associated enhancers.

Using embryonic heart organ culture and transgenic analysis, Huang et al. identified injury/regeneration‐related enhancers (Huang et al., 2012). These authors initially extracted evolutionarily conserved sequences that were associated with epicardial gene expression, and then investigated the activities using mouse embryonic heart organ culture, which potentially represents a developmental enhancer (Huang et al., 2012). These enhancers were validated based on their activities in injured hearts of reporter‐transgenic mice (Huang et al., 2012). These experiments showed that developmental enhancers are reused as injury/regeneration enhancers in adult tissues.

Enhancers associated with heart and caudal fin regeneration have been also identified in zebrafish (Kang et al., 2016). Kang et al. searched for genes that are induced in regenerating heart and caudal fin tissue, and found that *leptin b (lepb)* was highly expressed in these tissues. BAC transgenic analyses indicated that injury/regeneration‐associated enhancers of *lepb* are located within 150 kb of the *lepb* gene body. Moreover, comparisons of H3K27ac levels between uninjured and regenerating hearts further narrowed candidates to two enhancers located 7‐ and 3‐kb upstream of *lepb*. Finally, transgenic analysis of candidate enhancer elements showed that the element located 7‐kb upstream of *lepb* had enhancer activity in myocardial and epicardial tissues after cardiac injury and in regenerating caudal fin (Figure 2b). Histone H3 has four variants (H3.1 H3.2, H3.3 and H3.4) and previous studies show that the histone H3.3 variant is deposited at nuclease‐hypersensitive sites (Mito, Henikoff, & Henikoff, 2005; Mito, Jorja, & Teven, 2007). Accordingly, profiling of elements that occupy cardiomyocyte‐specific histone H3.3 in regenerating hearts identified a candidate regeneration enhancer. Subsequent transgenic reporter analyses showed that the H3.3‐enriched elements in regenerating hearts have enhancer activities after injury (Goldman et al., 2017). Chromatin remodeling is a known consequence of epigenetic modifications and is also essential to gene regulation. SWI/SNF chromatin remodeling complexes generally comprise 9–12 subunits with a core ATPase of SWI/SNF related, matrix associated, actin dependent regulator of chromatin, subfamily a, member 2 (Smarca2, alias name: Brahma homolog (Brm)) and Smarca4 (alias name: BRM/SWI2‐related gene 1(Brg1)). Wilms’ tumor 1 (Wt1) is a regulatory gene of epicardium‐derived cells that contributes to cardiovascular cell types and is activated in adult epicardium after myocardial infarction (Vieira et al., 2017). In addition, Brg1 and Brm expression in the epicardium is increased after myocardial infarction (Vieira et al., 2017). Comparisons of the Wt1 locus between humans and mice revealed seven evolutionarily conserved regions (ECRs). Moreover, SWI/SNF complexes, with C/EBP TFs, bind to ECRs in injured adult hearts, but not in intact hearts (Vieira et al., 2017). Thus, with chromatin remodeling complexes, C/EBP regulates *Wt1* expression in the adult epicardium after myocardial infarction through the seven ECRs (Vieira et al., 2017).

Thus, vertebrate injury/regeneration‐associated enhancers have been identified by narrowing down putative enhancers based on genome‐wide information and then validating enhancer activities in transgenic animals. The ensuing activation mechanisms, however, remain poorly understood.

## 8 AMPHIBIAN: A MODEL SYSTEM FOR REGENERATION STUDY

The anuran amphibian *X. laevis* and the urodele amphibians *Notophthalmus viridescens*, *Ambystoma tigrinum*, and *Ambystoma mexicanum* have been used as model animals for regeneration studies, because compared with mammals, these amphibians have high regenerative capacity. Recently, the anuran amphibian *Xenopus tropicalis* (*X. tropicalis*) and the urodele amphibian *Pleurodeles waltl* were investigated as novel model animals for regeneration studies (Liao et al., 2017; Elewa et al., 2017; D. Muñoz, Castillo, Henríquez, & Marcellini, 2018). Regenerative capacity of adults is the chief difference between anuran and urodele amphibians. Specifically, anuran amphibians experience progressive declines in regenerative capacities as in mammals, whereas urodele amphibians maintain their capacity to regenerate limbs even during adulthood (Yun, 2015; Tanaka, 2016; Haas & Whited, 2017). Experimentally induced metamorphosis in adult *Ambystoma mexicanum*, however, reduces regenerative ability, suggesting that the declining regenerative capacities with maturation also occurs even in urodele amphibians (Monaghan et al., 2014). Thus, comparisons of regenerative capacities of different species powerfully reveal the molecular mechanisms behind regeneration, although knowledge of differences between natural and experimentally induced maturation is crucial for the understanding of declines in regenerative ability.

Precise genomic information is necessary to analyze the functions of noncoding regions, and higher‐quality genomic information has been established for *X. laevis* and *X. tropicalis* than for other amphibians (Hellsten et al., 2010; Session et al., 2016). Therefore, the anuran amphibians *X. tropicalis* and *X. laevis* are poised for explorations of the roles of *cis*‐regulatory sequences in regeneration, despite being diploid and allotetraploid, respectively. This difference in ploidy indicates that, compared with *X. tropicalis*, the *X. laevis* genome contains almost twice the number of developmental genes, thus doubling the number of genes to consider in studies of the activation mechanisms of enhancers. Hence, with advantages of diploidy, *X. tropicalis* is the choice of organism for studies of noncoding DNA regions, yet because *X. laevis* has been used as a model animal for a long time, accumulated knowledge is highly advantageous for regeneration studies. In addition, transgenic lines for live imaging studies and systems for high‐throughput transgenesis have been established for *X. laevis* (Kroll & Amaya, 1996; Ogino, Fisher, & Grainger, 2008). Therefore, combined approaches using *X. tropicalis* genomic information and *X. laevis‐*based transgenic systems offer the greatest potential for studies of *cis*‐regulatory mechanisms of regeneration.

## 9 ACTIVATION MECHANISMS OF REGENERATION SIGNAL‐RESPONSE ENHANCERS

Kidneys are indispensable for vertebrate homeostasis, and loss of this organ causes severe defects. In vertebrates complex pronephros, mesonephros, and metanephros kidney structures have evolved. Metanephros refers to adult kidneys in higher vertebrates, such as humans and mice, and mesonephros refers to adult kidneys in amphibians and fish (Desgrange & Cereghini, 2015). Pronephros is the simplest and earliest kidney form (Jones, 2005). Although humans, mice, amphibians, and fish have differing kidney types, they all depend on a similar functional unit, the nephron, (Lienkamp, 2016). In humans and mice, the nephron comprises a glomerulus, which acts as a filtering component, and a nephric tubule, which comprises the four basic proximal tubule, loop of Henle, distal tubule, and connecting tubule domains (Saxen, 1987). *X. laevis* and zebrafish also have similar structures to that of the mammalian nephron, suggesting that *X. laevis* and zebrafish are suitable for studies of renal regeneration (Kroeger & Wingert, 2014; Raciti et al., 2008). In zebrafish, transplantation of *lhx1a‐*positive or *six2*‐positive mesenchymal cells, which are considered stem cells, into adults with gentamicin‐induced kidney injury led to the reconstruction of functional nephrons (Diep et al., 2011). *X. laevis* can also regenerate functional pronephros, with restored albumin uptake after mechanical loss of proximal tubules (Caine & Mclaughlin, 2013). Zebrafish use kidney stem cells to regenerate functional nephrons, whereas *X. laevis* appear to use the remaining tubule cells (Diep et al., 2011; Caine & Mclaughlin, 2013). In any case, zebrafish and *X. laevis* regenerate fully coiled and functional nephric tubule architecture after severe damage (Diep et al., 2011; Caine & Mclaughlin, 2013).

Mammals have limited capacity to regenerate the nephron, despite the presence of mature tubular epithelial cells that can regenerate following acute kidney injury. These epithelial cells of the nephron dedifferentiate into mesenchymal‐like cells and then migrate into regions of cell damage. Mammals, therefore, can only reconstitute epithelial cells after kidney injury (Maeshima et al., 2014).

Given the differences in regenerative capacity among vertebrates, it was likely lost over the course of evolution in some animals (Bely, 2010; Bely & Nyberg, 2010). As discussed above, many developmental genes that are evolutionarily conserved among vertebrates can be reactivated during regeneration. Thus, we hypothesized that highly regenerative animals carry genetic enhancers for regeneration, and that these enhancers are evolutionarily conserved among species. We also suggest that animals with low regenerative capacity, such as mammals, lost many regenerative enhancers.

**Figure 3:**
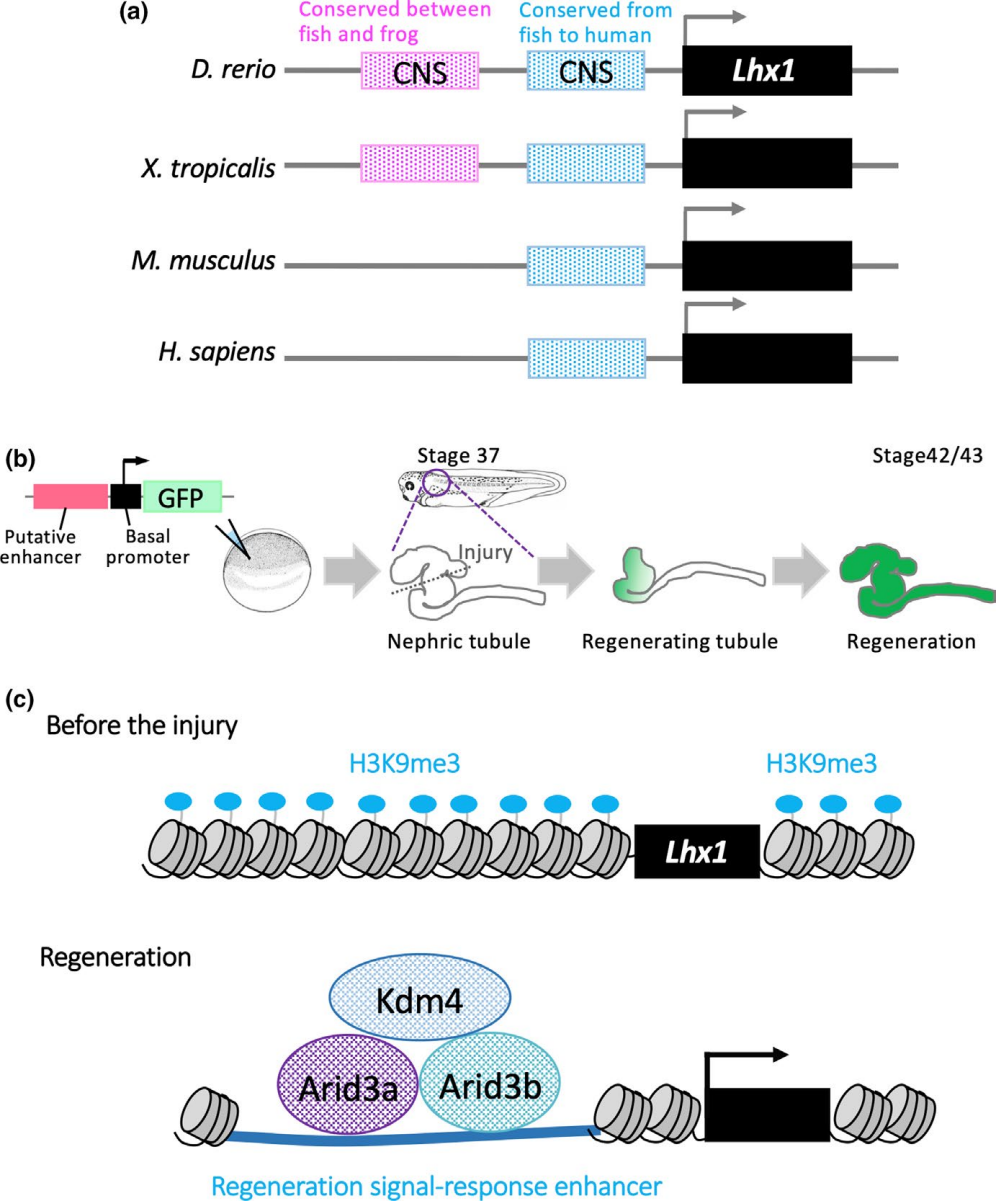
(a) Extraction of candidate enhancers for *Lhx1* using evolutionarily conserved noncoding sequences; the magenta box indicates the noncoding evolutionarily conserved sequence (CNS) between frogs and fish. The blue box indicates the CNS among vertebrates. (b) Identification of regeneration‐related enhancers for frog nephric tubules; nonmosaic founder frogs are generated by injecting reporter DNA and sperm nuclei into unfertilized eggs. Functional enhancers in regenerating tissues can be identified using founder transgenic animals. (c) Mechanisms of activation of regeneration signal‐response enhancers (RSREs); with the H3K9me3 demethylase–Kdm4 complex, Arid3a binds to RSREs and reduces H3K9me3 levels, thereby promoting the expression of *Lhx1* during regeneration of nephric tubules

To identify enhancers of kidney regeneration, we focused on two categories of CNS. The first group of sequences are evolutionarily conserved between *X. tropicalis* and zebrafish, which have high regenerative capacity, and are assumed to function as enhancers for regeneration. In the second group, sequences are evolutionarily conserved among vertebrates and may not be used for tissue regeneration, but may contribute as developmental enhancers for common traits among vertebrates (Figure 3a).

*X. laevis *provide an excellent system for identifying *in vivo* functions of *cis*‐regulatory sequences, because nonmosaic founder transgenic frogs can be generated by injecting reporter DNA with sperm nuclei into unfertilized eggs (Kroll & Amaya, 1996; Ogino et al., 2008; Ochi et al., 2012; Suzuki, Hirano, Ogino, & Ochi, 2015; Ochi, Suzuki, et al., 2017; Ochi, Kawaguchi, et al., 2017). Due to the challenges of demonstrating regenerative functions of enhancers *in vivo*, few regenerative enhancers have been identified to date. In contrast, nonmosaic founder reporter‐transgenic frogs offer convenient models for identifying functional enhancers in regenerating tissues (Figure 3b). We identified enhancers at the *Lhx1* locus that are activated in regenerating frog tissue (Suzuki, Hirano, Ogino, & Ochi, 2019). Although noncoding elements that are conserved between highly regenerative species have enhancer activities, these elements did not have strong enhancer activities in the regenerating amphibian nephric tubules (Suzuki et al., 2019). Instead, noncoding elements that are conserved between fish and humans function as enhancers in regenerating nephric tubules (Suzuki et al., 2019). Hence, mammals with limited regenerative abilities may retain regeneration signal‐response enhancers (RSREs) in their genomes. Further studies of the transcriptional mechanisms behind reactivation of amphibian developmental gene expression showed that Arid3a, which is a member of the AT‐rich interaction domain family, forms complexes with the H3K9me3 demethylases Kdm4a (previously named Jumonji domain containing 2A) and modulates H3K9me3 levels on RSREs (Figure 3c). Moreover, conditional knockdown of Arid3a using photo‐morpholino oligonucleotides inhibited nephric tubule regeneration, and conditional induction of Arid3a using heat shock promoter increased outgrowth of nephric tubules from distal tubules, which do not have the proliferative activity (Suzuki et al., 2019). Thus, Arid3a contributes to regeneration of nephric tubules by decreasing H3K9me3 on RSREs. Taken together, combinational approaches that narrow down candidate enhancers based on the conservation of noncoding sequences between divergent vertebrate species, and validation of enhancer activities in regenerating tissue using *X. laevis* transgenic systems, showed that regenerative genes and enhancers are evolutionarily conserved among vertebrates.

## 10 PERSPECTIVES AND FUTURE QUESTIONS

**Figure 4:**
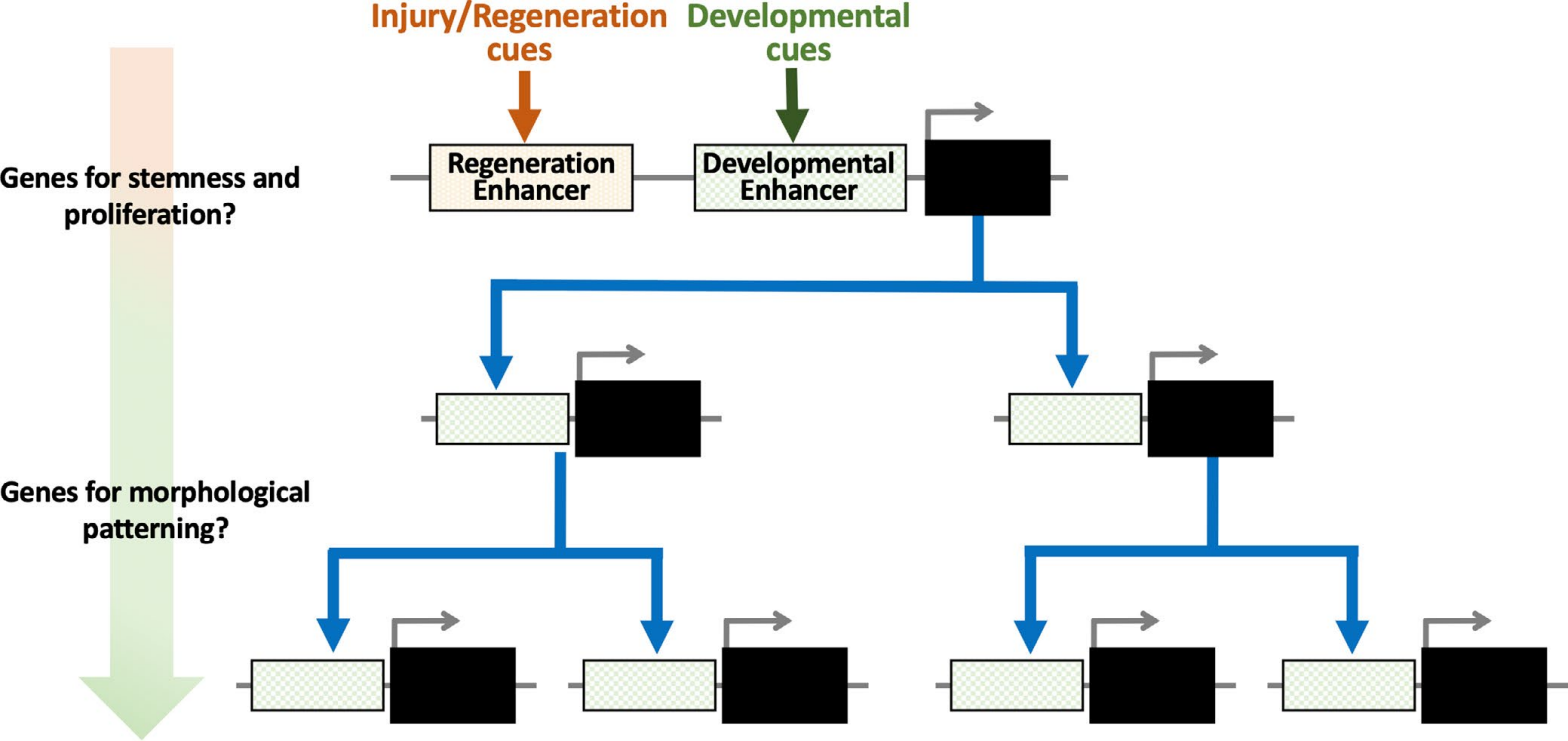
Injury/regeneration‐specific enhancers and developmental enhancers; genes that initiate the developmental cascade may have injury/regeneration‐specific enhancers. In contrast, genes located downstream of the cascade may reuse developmental enhancers for regeneration

Many previous studies show that numerous developmental genes are expressed during tissue regeneration. It is also known that these developmental genes are evolutionarily conserved among vertebrates. Because re‐expression of developmental genes requires *cis*‐regulatory sequences, such as promoters and enhancers, the mechanisms behind activation of regenerative enhancers are particularly important for understanding regeneration. Recent studies show that some genes possess enhancers that primarily function in regenerating tissue not in development, while others reuse developmental enhancers for regeneration (Kang et al., 2016; Suzuki et al., 2019; reviewed in Rodriguez & Kang, 2019; Yang & Kang, 2019; Figure 4). Moreover, because transcriptional cascades of regeneration basically recapitulate developmental processes, genes with injury/regeneration‐specific enhancers may function as triggers of regeneration at the start of the developmental gene cascade (Figure 4). In contrast, genes that reuse developmental enhancers for regeneration might be located downstream of gene regulatory networks (Figure 4). Further studies of relationships between genes with injury/regeneration‐specific enhancers and genes that reuse developmental enhancers may indicate *cis*‐regulatory mechanisms that are characteristic of regeneration and resemble the developmental process.

The present studies show that injury/regeneration‐related enhancers are evolutionarily conserved among vertebrates (Suzuki et al., 2019), yet limited regenerative capacity is clear among mammals. Other studies suggest that such regeneration‐related enhancers in mammals are epigenetically silenced, but little epigenomic data are available for highly regenerative animals, compared with that for mammals, and it remains unclear whether enhancers in highly regenerative animals are not silenced by epigenetic modifications during entire lifespans. Moreover, if this is the case, the molecular mechanisms behind the nonsilencing enhancers remain unclear. Further comparisons of epigenetic controls on the expression of regeneration‐related enhancers between different taxa and throughout the lifespan are required. In addition, identification of silencer elements will improve our understanding of the molecular basis of regeneration.

## AUTHOR CONTRIBUTIONS

H.O. and N.S. discussed the topics to be reviewed. H.O. wrote the manuscript. H.O. and N.S. commented on the manuscript.
